# Brucellosis in livestock: First study on seroepidemiology, risk factors, and preventive strategies to manage the disease in Famenin, Iran

**DOI:** 10.14202/vetworld.2022.2102-2110

**Published:** 2022-08-31

**Authors:** Maryam Adabi, Salman Khazaiee, Ali Sadeghi-Nasab, Saeed Alamian, Mohammad Reza Arabestani, Zahra Valiei, Jamal Gharekhani

**Affiliations:** 1 Brucellosis Research Center, Hamadan University of Medical Sciences, Hamadan, Iran; 2 Research Center for Health Sciences, Hamadan University of Medical Sciences, Hamadan, Iran; 3 Department of Clinical Sciences, Faculty of Veterinary Sciences, Bu-Ali Sina University, Hamedan, Iran; 4 Department of Brucellosis, Razi Vaccine and Serum Research Institute, Agriculture Research, Education and Extension Organization, Karaj, Iran; 5 Department of Microbiology, Faculty of Medicine, Hamadan University of Medical Sciences, Hamadan, Iran; 6 Department of Microbiology, Faculty of Basic Sciences, Hamadan Branch, Islamic Azad University, Hamadan, Iran; 7 Department of Laboratory Sciences, Central Veterinary Laboratory, Iranian Veterinary Organization, Hamedan, Iran

**Keywords:** animal, brucellosis, endemic, prevention, risk factors

## Abstract

**Background and Aim:**

Brucellosis is an infectious disease in humans and livestock. The disease is endemic in many regions of Iran, for example, Hamedan Province. Knowledge of infection rate and associated risk factors is essential to control and prevent the disease. The study aimed to estimate the prevalence of brucellosis and associated risk factors in cattle, sheep, and goats in Famenin, Hamedan Province, West of Iran.

**Materials and Methods:**

Blood samples of 1758 animals (1470 sheep, 190 goats, and 98 cattle) were obtained in different rural regions of Famenin. The samples were evaluated to detect of *Brucella*-antibodies using rose Bengal plate test (RBPT), Wright standard tube agglutination test (SAT), and 2-Mercapto-Ethanol (2-ME) techniques. The risk factors associated with brucellosis such as age, gender, history of vaccination against brucellosis, and abortion history in animals were evaluated. In the sampling process, the critical gaps related to the distribution of brucellosis in the herds and regions are identified for designing the strategies to prevent and control the disease.

**Results:**

About 6.88% and 89.31% of animals had a history of abortion and vaccination against brucellosis, respectively. Most of the animals were female (92.49%) and in the range of 2–3 age old (39.8%). The antibodies to the *Brucella*-infection in animals were 2.73% with RBPT and 1.30% with SAT and 2-ME. The prevalence of brucellosis was detected 1.3% among individual animals and 11% among herds. This rate was 1.43% for sheep and 1.05% for goats, with no significant statistical difference. No seropositive case was detected in cattle samples using RBPT, STAT, and 2-ME. The highest rate of brucellosis (6.25%) was detected in Emamzadeh-Pirnahan region (22.2% goats and 5.6% sheep). In sheep, most cases of the disease were in 3–4 age-old group (1.92%), animals without a history of abortion (1.58%), and without a history of vaccination against brucellosis (2.80%). Furthermore, 5.94% of males and 1.11% of females were detected positive for brucellosis (p < 0.001). The chance of brucellosis in rams was 5.6 folds higher than in others (odds ratio = 5.64). Brucellosis in goats was detected 2.94% and 1.89% in the age groups <1 and 2–3 year-old. Furthermore, 1.22% of females and 1.34% of animals without a history of abortion were positive. Brucellosis was found in 0.61% of vaccinated and 3.85% of non-vaccinated goats. Except for gender in sheep, no significant statistical correlation (p > 0.05) was observed between prevalence of brucellosis and risk factors. In farmers, low level of information about the transmission and also control and preventive methods of the disease was dominant. Consumption of traditional and unpasteurized dairy products is also very common in the studied regions.

**Conclusion:**

This is a comprehensive evaluation of animal brucellosis parallel to humans’ cohort study in the Famenin region for the first time. Although the rate of brucellosis in animals is low in the region, explaining the risk factors to farmers, mass vaccination, regular screening of animals, and culling the positive animals are very important for controlling and reducing the disease in the region.

## Introduction

Brucellosis is one of the most important zoonotic diseases in the world, especially in the Middle East and North Africa [1]. The disease is prevalent in many places, especially in developing countries. It has been a great impact on public health and economy [2]. The World Health Organization (WHO) reports that more than 500,000 new cases of brucellosis are recognized in humans on a global scale annually [3]. Brucellosis caused by the various species of *Brucella*, an intracellular coco-bacilli Gram-negative bacteria, can be transmitted to humans through consumption of unpasteurized dairy products and/or direct contact with secretory materials of infected animals [4]. *Brucella melitensis* and *Brucella abortus* are common species presenting a risk to humans and livestock. *Brucella pinnipedialis*, *Brucella ceti*, and *Brucella microti* are newly described species isolated from seals, dolphins, and wolves [5]. Brucellosis has multiple manifestations in the hosts and can affect the different organs in human. In animals such as livestock, *Brucella* has a high tendency to infect genital organs. The main clinical features in livestock is abortion, reproductive failures (in both gender), and reduction of milk production, which is a top manifestation in domestic animals [6]. Esmaeili [7] reports that 20% loss of milk production, 2–3 fold abortion, and 10% of infertility happen due to brucellosis in animals. Abortion related brucellosis is estimated to be between 30% and 80% in dairy herds with traditional management [8]. There are many reports of human disabilities, economic losses, and obligatory culling due to *Brucella*-infection in animals on a global scale. The economic losses due to these complications are very significant for farmers and at the national level in the countries where the disease is endemic. Hence, it needs careful attention of authorities [1]. There is a correlation between brucellosis in livestock and humans [6]. No treatment is recommended for brucellosis in animals; thus, culling is the best strategy to stop the transmission of the disease from animals to humans [9].

Brucellosis is an endemic zoonotic disease in many parts of Iran [10]. In Iran, the seven-year incidence rate of brucellosis (2011–2018) was 21.78% (95% confidence interval [CI]: 21.66–21.91%) [11]. Mirnejad *et al*. [3] reported the annual incidence of brucellosis is 0.001% in Iran. Unlike in many countries, the disease is a public health problem in Iran [7]. This problem is common in Hamedan Province, West of Iran, with an incidence rate of 31–41 per 100,000 people [12]. Consumption of non-pasteurized dairy products, especially from ewes, is common in most rural areas, so the prevalence of brucellosis is high compared to urban areas. Brucellosis usually occurs in grasslands at a moderate elevation and during spring, where sheep and goats are the dominant livestock [13]. In studies about Hamedan Province, 8.1% of veterinarians, 15% of abattoir staff, and 17% of butchers were reported to be *Brucella*-infection positive [14]. In addition, according to Gharekhani *et al*. [15], Gharekhani and Sazmand [16], 3.3% of dogs had antibodies to *Brucella*-infection. Interestingly, no antibody was detected in the studied horses.

For a comprehensive evaluation of brucellosis in the animal population of Famenin, Hamedan Province, we designed a project parallel to the human cohort study in this area [17, 18]. This study aimed to estimate the prevalence and risk factors of brucellosis in sheep, goats, and cattle in this region.

## Materials and Methods

### Ethical approval

The study was approved by the ethics committee of Hamedan University of Medical Sciences (ID: IR.UMSHA.REC.1398.4).

### Study period and location

**Figure-1:**
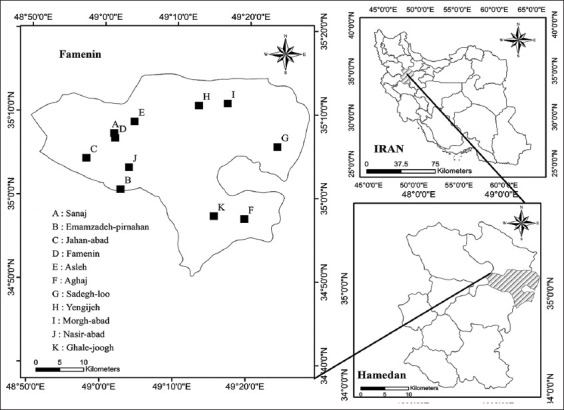
The geographic map and distribution of sampling regions (n = 11, A-K) in Famenin, Hamedan Province, West of Iran [Source: www.ncc.gov.ir].

The study was conducted from September 2020 to March 2021 in Famenin. Famenin is a region in the North-East of Hamedan province. Hamedan province, with an area of 19,546 km2 (34.77° N and 48.58° E) is located in the West part of Iran (Figure-1). The main occupation of the people in region is agriculture and animal husbandry. Much of animal husbandry is traditional in rural regions, and usually there are both sheep and goats in the herds. There are 37,000 cattle, 4,000 goats, and 101,000 sheep in Famenin.

### Study design and sampling method

This study is part of a cohort project to estimate the prevalence of brucellosis and its risk factors among people and domestic animals in the Famenin region [17, 18]. Considering the estimated prevalence of 2.5% of animal brucellosis in this region, taking into account the 95% confidence interval and also the margin of error equal to 25% of p, the estimated sample size was obtained 1167 samples using the Cochran formula [19]. Moreover, due to the fact that we used cluster sampling in the sampling stages, so the design effect (DE = 1.5) was included in the estimation of the sample size and finally, 1750 animals were studied. The method of allocating the estimated sample size to the covered population units was in accordance with Table-1.

**Table-1:** The method of allocating the estimated sample size to the covered population units.

Animals	Total population	Proportion	Sample size (approximately 1% of livestock)
Sheep and goats	183000	95% of livestock	1660
Cattle	4850	5% of livestock	90
Total	187850	-	1750

The size of clusters was considered as 160 (150 sheep and goats and 10 cattle) and therefore, 11 clusters were selected according to the livestock population in each area. The number of samples from each location is shown in Table-2. The number of clusters and distribution in Famenin was based on the proportional number of livestock in each location to the total livestock of Famenin. A total of 1758 jugular blood samples (1470 sheep, 190 goats, and 98 cattle) were obtained by disposable needles and Venoject tubes using regular simple cluster sampling from 109 herds of eleven locations (A-K) in the studied area (Figure-1).

**Table-2:** The rate of brucellosis based on serology from Famenin in different animals and sampling locations.

Sampling location	Sheep		Goat		Cattle		Total	
			
	No. of sample	Positive (%)	No. of sample	Positive (%)	No. of sample	Positive (%)	No. of sample	Positive (%)
Asleh	125	0	25	0	10	0	160	0
Emamzadeh-Pirnahan	141	8 (5.6)	9	2 (22.2)	10	0	160	10 (6.25)
Famenin	142	5 (3.5)	8	0	10	0	160	5 (3.1)
Ghale-joogh	134	3 (2.2)	16	0	10	0	160	3 (1.8)
Jahan-Abad	150	2 (1.3)	NS	10	0	160	2 (1.25)
Morgh-Abad	131	0	19	0	13	0	163	0
Nasir-Abad	144	2 (1.3)	6	0	10	0	160	2 (1.25)
Sadeghloo	129	0	21	0	NS	150	0
Sanaj	134	0	16	0	10	0	160	0
Yengijeh	98	0	52	0	15	0	165	0
Aghaj	142	1 (0.7)	18	0	NS	160	1 (0.62)
Total	1470	21 (1.43)	190	2 (1.05)	98	0	1758	23 (1.3)

NS=No sample

**Figure-2:**
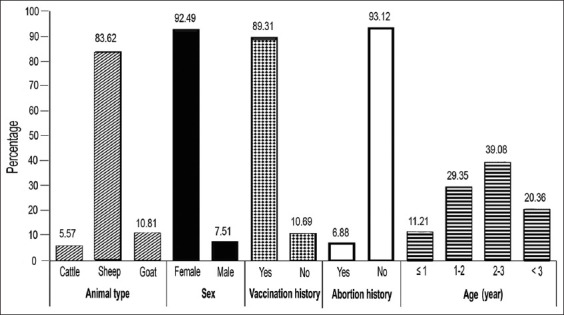
Demographic characteristics associated with sampled animals in Famenin.

In the sampling process, the demographic information of animals and the risk factors associated to brucellosis such as age (<1, 1–2, 2–3, and 3–4 year age-old groups), gender (Male or Female), history of vaccination against brucellosis (Yes or No), and abortion history (Yes or No) in animals was recorded after inserting an era tag (Figure-2 and Table-3). In addition, the critical gaps related to the distribution of brucellosis in the herds and regions are identified for designing the strategies to prevent and control the disease.

**Table-3:** Brucellosis in sheep and goats from Famenin in different evaluated risk factors.

Animals	Risk factors	Sample No. (%)	Positive (%)	Statistical analysis	Odds Ratio (OR)***
Sheep (n = 1470)	Age				
	<1	158 (10.75)	2 (1.27)	Pearson Chi-square (3) = 0.76 p = 0.86**	Reference
	1–2	460 (31.29)	7 (1.52)		1.21 (0.25, 5.86)
	2–3	592 (40.27)	7 (1.18)	0.93 (0.19, 4.54)
	3–4	260 (17.69)	5 (1.92)	1.53 (0.29, 7.98)
	Sex				
	Male	101 (6.87)	6 (5.94)	Pearson Chi-square (1) = 15.43 p < 0.001**	Reference
	Female	1369 (93.13)	15 (1.11)		5.64 (2.13, 14.86)
	Vaccination				
	Yes	1327 (90.27)	17 (1.28)	Pearson Chi-square (1) = 2.10 p = 0.15**	Reference
	No	143 (9.73)	4 (2.80)		2.22 (0.74, 6.68)
	History of Abortion				
	Yes	107 (7.82)	1 (0.93)	Pearson Chi-square (1) = 0.2 p = 0.60*	Reference
	No	1262 (92.18)	20 (1.58)		1.57 (0.21, 11.87)
Goat (n = 190)	Age				
	<1	34 (17.89)	1 (2.94)	Pearson Chi-square (3) = 2.61 p = 0.31*	-
	1–2	68 (35.79)	0		-
	2–3	53 (27.90)	1 (1.89)		-
	3–4	35 (18.42)	0		-
	Sex				
	Male	26 (13.68)	0	Pearson Chi-square (1) = 0.32 p > 0.99*	-
	Female	164 (86.32)	2 (1.22)		-
	Vaccination				
	Yes	164 (86.32)	1 (0.61)	Pearson Chi-square (1) = 2.26 p = 0.26*	-
	No	26 (13.68)	1 (3.85)		-
	History of Abortion				
	Yes	15 (9.15)	0	Pearson Chi-square (1) = 0.17 p > 0.99*	-
	No	149 (90.85)	2 (1.34)		-

* Fisher exact test,

** Chi-square test,

*** We could not estimate OR for goat due to low number of positive cases

### Serology

The sera samples were separated after whole blood centrifugation (1400× *g* for 12 min) and kept under −18°C until the examination [20]. At first, all samples were screened by rose Bengal plate test (RBPT). Then, the positive samples were re-evaluated using Wright standard tube agglutination test (SAT) and 2-Mercapto-Ethanol (2-ME) techniques. The final interpretation of serological results (positive or negative for brucellosis) was performed according to the protocol presented in Table-4 [20].

**Table-4:** The guideline for interpreting the results of brucellosis serology tests in livestock in Iran (Iranian Veterinary Organization protocol).

Animals	Serology			Brucellosis result

	RBPT	SAT	2-ME
Sheep and goat	Positive	≥4/40	Each titer of antibody	Positive
		≤3/40	≥1/20	Positive
			≤4/10	Negative
Cattle		≥1/160	Each titer of antibody	Positive
		4/40–4/80	≥4/40	Positive
		≤1/20	≤1/20	Negative

RBPT=Rose Bengal plate test, SAT=Standard tube agglutination test, 2ME=2-Mercapto-Ethanol

### RBPT

An equal level (30 mL) of RBPT antigen (Vaccine and Serum Research Institute of Razi, Karaj, Iran) and sera samples were mixed by a disposable applicator on a white ceramic tile and shaken for 4 min. A positive result was reported after observing the pink agglutination [20–22].

### Standard tube agglutination test

Briefly, 0.8 mL of phosphate buffer saline (PBS) was dispensed to the first agglutination tube. Then, 0.5 mL PBS was applied to the 2^nd^, 3^rd^, 4^th^, and 5^th^ tubes. In the next stage, 0.2 mL of sera sample was added to the 1^st^ tube and shaken correctly. Serial dilution was carried out by pipetting 0.5 mL of the mixture of the 1^st^ tube to 2^nd^ and then to other tubes. In the end, 0.5 mL of materials from the 5^th^ tube was thrown away. Then, 0.5 mL of Wright antigen with 1/10 concentration (Vaccine and Serum Research Institute of Razi) was added to the contents of all test tubes and incubated at 37°C for 24 h after shaking. The agglutination titers were recorded using guidelines [20–22].

### 2-Mercapto-Ethanol

This technique is similar to SAT. A volume of 0.3 mL and 0.5 mL of PBS were dispensed in the first and other tubes, respectively. Then, 0.2 mL of sera sample was added to the first tube. In the experiment, we added 0.5 mL of 2-ME solution (Merck, Germany: 68 μl of 2-ME solution in 5 mL distilled water) to the first tube and shaken it completely and incubated it at 37°C for 1 h. Serial dilution was carried out by pipetting 0.5 mL of the mixture of the 1^st^ tube to 2^nd^ and then to the following tubes, respectively. In the end, 0.5 mL of materials from the 5^th^ tube was transferred out. Then, 0.5 mL of Wright antigen with 1/10 concentration (Vaccine and Serum Research Institute of Razi) was added to the contents of all test tubes and incubated at 37°C for 24 h after shaking. The agglutination titers were recorded using guidelines [20–22].

### Statistical analysis

The qualitative data were summarized with frequencies and percentages and the quantitative variables with the mean and the standard deviation. The association between demographic variables of the animals with their disease status was assessed by Chi-square test. Moreover, the effect of gender on brucellosis disease was assessed using the logistic regression model. Data were analyzed using Stata software version 14 (www.stata.com/stata14). The significant level was considered <0.05.

## Results

### Prevalence rate

About 6.88% and 89.31% of animals had a history of abortion and vaccination against brucellosis, respectively. Most of the animals were female (92.49%) and in the range of 2–3 age old (39.8%) (Figure-2). Using screening RBPT, zero, 3.13% (46/1470) and 1.05% (2/190) of sera samples were positive in cattle, sheep, and goats, respectively (Table-5). In regard to inserted guideline in Table-1, the overall prevalence of brucellosis in animals was detected 1.3% (95% CI: 0.8–1.8%) in individuals and 11% (12/109, 95% CI: 5.2–16.8%) in herds (Table-5). This rate was calculated as 1.43% (21/1470, 95% CI: 0.8–2.03%) for sheep and 1.05% (2/190, 95% CI: 0–2.5%) for goats. No seropositive case was detected in cattle samples using serology (Tables-2 and 5). The prevalence of brucellosis in sheep was higher than in goats; but there was no significant difference between positive samples of sheep and goats (p = 0.67). Antibodies to *Brucella*-infection in animals were 2.73% using RBPT and 1.30% using SAT and 2-ME (Table-5). The prevalence rate regarding animals and different serologic techniques is summarized in Table-5.

**Table-5:** Seroprevalence of brucellosis regarding animals, gender, and diagnostic methods.

Animals	Sample No. (%)			RBPT		SAT and 2-ME		Final Positive (%)
		
	Total No. (%)			Positive No. (%)	Negative No. (%)	Positive No. (%)	Negative No. (%)	
Sheep	1470 (83.62)	♀	1369 (93.2)	32 (2.34)	1337 (97.66)	15 (1.11)	1354 (98.90)	21 (1.43)
		♂	101 (6.8)	14 (13.90)	87 (86.14)	6 (5.94)	95 (94.06)	
Cattle	98 (5.57)	♀	93 (94.9)	0	93 (100)	0	93 (100)	0 (0)
		♂	5 (5.1)	0	5 (100)	0	5 (100)	
Goat	190 (10.81)	♀	164 (86.32)	2 (1.22)	162 (98.78)	2 (1.22)	162 (98.78)	2 (1.05)
		♂	26 (13.68)	0	26 (100)	0	26 (100)	
Total	1758 (100)			48 (2.73)	1710 (97.27)	23 (1.30)	1735 (98.7)	23 (1.30)

RBPT=Rose Bengal plate test, SAT=Standard tube agglutination test, 2ME=2-Mercapto-Ethanol

There were no cattle in some of the sampling locations (Table-2). In addition, there was not any confirmed brucellosis in locations of Asleh, Morgh-Abad, Sadeghloo, Sanaj, and Yengijeh. The highest rate of abortion was seen in Morgh-Abad and Yengijeh regions, with 25 cases in each location. The minimum belonged to Ghale-joogh and Sadeghloo regions, with no reported cases of abortion. Furthermore, most cases of abortion were seen in animals aged 2–3 years old. The highest rate of brucellosis (6.25%) was detected in Emamzadeh-Pirnahan region (22.2% goats and 5.6% sheep) (Table-2).

### Risk factors

Prevalence rate of brucellosis with different evaluated risk factors and also animal type is presented in Table-3.

### Sheep

In the examination of 1470 sheep samples, most cases of brucellosis were in 3–4 age-old group (1.92%, 5/260), animals without a history of abortion (1.58%, 20/1262). and without a history of vaccination against brucellosis (2.80%, 4/143). About 5.94% (6/101) of males and 1.11% (15/1369) of females were detected to be positive for brucellosis (p < 0.001). The chance of brucellosis in rams was 5.6 folds (95% CI: 2.13–14.86%) higher than in others (odds ratio [OR] = 5.64). Except for gender in sheep, no significant statistical correlation (p > 0.05) was observed between the prevalence of brucellosis and the presented variables (Table-3).

### Goats

In regard to serology, two out of 190 (1.05%) samples were positive for brucellosis. We could not calculate OR or the risk factors due to a low number of positive cases. Brucellosis was detected only in the age groups of less than 1 (2.94%, 1/34) and 2–3 year-old (1.89%, 1/53), female animals (1.22%, 2/164), and animals without a history of abortion (1.34%, 2/149). About 86.32% of goats had a history of vaccination against brucellosis. However, brucellosis was found in 0.61% (1/164) of vaccinated and 3.85% (1/26) of non-vaccinated animals. There were no significant statistical differences in the prevalence of brucellosis in goats and evaluated risk factors (p > 0.05, Table-3).

### Prevention gaps

Most of farmers in the studied regions did not have enough information about the transmission and also control and preventive methods of the disease. Consumption of traditional and unpasteurized dairy products is also very common.

## Discussion

The most important concern about brucellosis is health. It is also responsible for significant economic losses in the livestock industry, especially in endemic areas. Brucellosis is one of the most important infectious diseases in Iran [1]. All domestic and wild animals as well as pets, act as the reservoir of transmitting the *Brucella*-infection in humans [6]. Brucellosis is transmitted through both vertical and horizontal ways in animals. There is a high concentration of *Brucella* in the vaginal discharge of infected animals [23]. Risk factors and epidemiological data on brucellosis are needed for designing a comprehensive program to prevent and control it in any region [20]. In Iran, most sheep and goats are bred traditionally with few hygienic guidelines. Farmers use mobile and semi-mobile herd management practices and move periodically among different pasture areas and seasonally. In some countries with a high incidence of brucellosis, the frequent and unrestricted transfer of animals, especially within the national and international borders, poses a problem for the control of the disease. Therefore, international collaborations are needed to improve border security. Furthermore, in this region, the trade of live animals is much more common than the trade of animal products due to religious slaughtering practices and Muslim festivals such as Hajj, Ramadan, Eid ul-Fitr, and Eid ul-Adha. Animal transferring and slaughtering during this time result in increased direct contact with animals, posing a risk of transmitting zoonotic diseases to humans.

There are different reports on animal brucellosis in Iran. With regard to the animal population in Iran, much work has been concentrated on cattle followed by sheep, goats, camel, and buffalo. Furthermore, the species of *B. melitensis*, *B. abortus*, and co-infection of *B. melitensis* and *B. abortus* are more dominant, respectively [1]. Initially, the rate of brucellosis among Iranian cattle was reported to be 17.6% [24]. Recent study [22] shows that seroprevalence of cattle brucellosis in different regions of Iran is 5.6%, 3.9%, and 4.9% using RBPT, SAT, and enzyme-linked immunosorbent assay, respectively. In addition, the prevalence of brucellosis in animals in rural areas is higher than peri-urban areas [22]. In a meta-epidemiological investigation by Dadar *et al*. [1], brucellosis was calculated to affect 10.18% of Iranian livestock; this rate was 2.2–5.7% in Hamedan Province. Regarding animal species, brucellosis rate is reported 14.66% in cattle, 12.83% in sheep, 11.97% in buffalo, 4.34% in goats, and 3.39% in camels. The infection rate in animals with clinical manifestations (38.65%) is significantly higher than in healthy animals (8.38%).

The RBPT, a rapid, simple, low-cost, and user-friendly method, is used for screening and detecting the antibodies to *Brucella*-infection such as immunoglobulin (Ig) M, IgG, and IgA. SAT and 2-ME techniques are applied routinely to detect the titer of antibodies in non-vaccinated and vaccinated animals, respectively. In serological tests, SAT remained popular and is used on a global scale [8]. We also used these serological methods based on previous studies. Using serological methods, we detected 1.3% and 11% of brucellosis in individual animals and herds, respectively. This rate was reported 1.43% and 1.05% for sheep and goats, respectively. In the cohort study conducted in Famenin, antibodies to *Brucella*-infection were 6.6% and 3.5% using SAT and 2-ME techniques, respectively [18]. About 43.4% of the positive cases were confirmed by molecular tools and 28.5% were positive for the species of *B. melitensis* and *B. abortus* [17]. In this work, contact with domestic animals and consumption of non-pasteurized dairy products, especially curd, were strong risk factors for brucellosis [2].

In a serology work from southern Iran [25], 3.36% of cattle and 3.27% of small ruminants (sheep and goats) were positive for brucellosis; this rate was 27% in sheep and goats at herd-level. In ZareBidaki *et al*. [10] study from Eastern Iran, 23% (23/100) of livestock were positive using SAT and 2-ME methods. According to Sharifi *et al*. [26], 2.7% of goats and 3.5% of sheep were positive for brucellosis in southeastern Iran. In a similar study in Chinese goats, 3.9%, 4.45%, and 86.67% of animals were seropositive using RBPT, SAT, and PCR methods, respectively [27]. Furthermore, in a meta-analysis work from China, 1.7% of animals were positive for brucellosis [6]. Furthermore, bovine brucellosis was reported 1.3–5.6% in African countries and 12% in India [28, 29]; whereas all of the sampled cattle were negative in our study. In neighboring countries, brucellosis was reported between 0.85% and 23.3% in animals [9]. In cattle, this rate was 7.2% in Kuwait, 18.1% in Jordan, 6.7% in Egypt, 2–20% in Turkey, and 3% in Iraq [9, 30–32]. Furthermore, the prevalence of brucellosis in small ruminants (sheep/goat) was 0–1.7% in Pakistan, 5.87–18.8% in Egypt, 22.2–45.4% in Jordan, and 2–4% in Yemen, 5.3–10.7% in United Arab Emirates, 15.6–3.9% in Saudi Arabia, and 15% in Iraq [9, 33–35]. Long borders with neighboring countries are a risk factor for increasing the disease in Iran.

In our study, the infection rate in sheep was more than in goats (p > 0.05). In a previous report from Hamedan Province [20], the brucellosis rate was 3% and 4.6% in sheep and goats, respectively. In addition, the infection rate in goats was significantly higher than in sheep (OR = 1.8). Reviriego *et al*. [36] and Suryawanshi *et al*. [37] results are different from the present study. In contrast, some of the researchers reported equal levels of the infection rate in sheep and goat flocks [38, 39].

The principal manifestation of brucellosis in livestock is abortion and also genitally disorders. Usually, shedding of the infected materials occurs after delivery. Therefore, the secretory materials are so harmful to public health [6] because of releasing highly infectious uterine secretions during the abortion and shortly after that could be an important source of contamination of the environment, grounds, and pastures that can spread the infection to other animals and humans [17]. About 0.93% of ewes with abortion history were positive for brucellosis; while all of the goats’ sample (n = 15) with a history of abortion was negative. In Iran, 14.2–25.7% of animals with a history of abortion were positive for *Brucella*-infection (p < 0.05) [1]. In Gharekhani *et al*. [20] investigation, the rate of brucellosis in livestock with an abortion history was significantly high (p < 0.05). Furthermore, the researchers confirmed a strong correlation between brucellosis and the incidence of abortion in animals such as sheep and goats [1]. An outbreak of abortion can lead to more incidence of *Brucella*-infection in animals and also farmers [10]. A relationship between abortion and prevalence rate of brucellosis was reported in different investigations [1]. In our work, the prevalence of brucellosis in animals with a history of abortion was not statistically significant. The finding highlighted the role of other risk factors (infectious and/or non-infectious) for abortion. Furthermore, farmers’ information may also be incomplete while obtaining the livestock history due to the traditional way of animal husbandry in the region.

In the case of brucellosis, the importance of producing preventive immunity in animals has always been considered. However, as the development of active immunity in humans is still in the experimental stages, and control of the disease in humans is possible by limiting the spread of infection in animals [6]. Vaccination is one of the most effective and practical methods for controlling brucellosis, which is applied successfully in most endemic areas in the world [4]. In our work, the brucellosis rate was less common in vaccinated livestock, parallel to the previous report in this area [20]. Fortunately, the rate of vaccination in Famenin was approximately 90% that might be better in the future. As seen in this study, vaccination could be one of the most important factors in decreasing the rate of infection among livestock. In Iran, vaccination with attenuated *B. melitensis* Rev.1 strain is used for immunization of sheep and goats and RB51 (attenuated *B. abortus*) for cows. The full and reduced doses were injected into the heifers or lambs and adults. They have been suggested as safe and effective approaches to eliminate brucellosis in ruminants [1]. In Emamzadeh-Pirnahan, one of the studied area, brucellosis was high compared to other locations. Therefore, preventive measures, especially vaccination of livestock against brucellosis in this area, should be prioritized compared to other regions.

In our findings, the prevalence of brucellosis in sheep aged 3–4 years was high compared to other groups. There was a different level of infection in age groups in sheep and goats with no significant statistical differences, similar to Teklue *et al*. [40] reports from Ethiopia. The results of age impact as a risk factor for brucellosis are high in relation to sample size and method of sampling. Negash *et al*. [39] believe that the infection rate in younger animals is high due to the low level of antibodies in the immune system. The chance of infection increases parallel to the age of animals; therefore, some researchers reported high infection in adult animals [41]. The age of animals was also reported as a significant risk option in the other works [10].

Brucellosis rate in rams was 5.6 times higher than in ewes in the Famenin region in accordance with a previous report in Hamedan Province [20]. Kiros *et al* [8] reportedthat brucellosis in female animals was 2.1 folds higher than in males. Furthermore, in a meta-analysis project from Iran, the prevalence rate in female animals (8.7–13.3%) was significantly more than in males (5.6–11.2%) [1]. In pregnant animals, Erythritol (an alcoholic material) is used in the fetus that provides suitable conditions for the growth of *Brucella* [20]. In Iran, male animals are less immune to brucellosis due to a lack of vaccination in some areas; therefore, males are more susceptible than females. Males are often used to mate, so they have a significant role in transmitting the infection.

In Gharekhani *et al*. [15] study from Hamedan, all of the milk samples from dairy farm tested were negative for *Brucella*-infection using molecular methods. Unlike this, wild and vaccinated of *Brucella* strains were isolated in milk samples of dairy farms in different regions of Iran [10]. However, vaccination against brucellosis must be the main goal in endemic regions because contact with livestock is a strong risk factor for human brucellosis [1, 22].

Detection of mixed infections of *B. abortus* and *B. melitensis* could be related to keeping different species of livestock together [10]. Farmers should avoid keeping different livestock together which is common in Iran. In ZareBidaki *et al*.’s [10] report, the presence of non-vaccinated and different types of animals on the farm, increase of animals’ age, incidence of abortion, and contact of livestock with wild animals were strong risk options for brucellosis. This may be due to differences in study design and protocols, type of samples and sample size, laboratory techniques, geographical regions, ecological factors, livestock density, and herd management [20]. To reduce brucellosis in the human community, the infection should be eliminated in reservoir hosts. In addition, using the vaccination and test-and-slaughter projects are so effective for this purpose.

To prevent the disease, the following 11 practical points were taught to the local farmers, who were mostly illiterate:

1. Vaccinate all of your livestock regularly
2. Keep livestock species (e.g., sheep and cattle) and different genders (i.e., rams and ewes) separately
3. Allocate specific pastures for each flock
4. Do not cross-move livestock between flocks
5. Test the rams for brucellosis regularly
6. Avoid slaughtering out of the registered abattoir
7. Remove the aborted/dead fetuses as soon as observed to avoid their consumption by carnivores, and destroy them preferably by incineration
8. Slaughter livestock at younger ages to decrease the infection risk
9. Gradually switch from traditional to industrial husbandry systems with higher hygienic measures
10. Avoid consumption of unpasteurized milk and dairy products
11. Cull the infected animals from the herds.

## Conclusion

This was a comprehensive evaluation of animals’ brucellosis parallel to humans’ cohort study in the region for the first time. We developed a program to cull positive animals in all of the sampled locations to cut the chain of infection transmission. The rate of brucellosis in animals was low in comparison to previous reports. Furthermore, cattle have no significant role in transmitting the infection to humans in the area. Educating farmers to learn about a better definition of risk factors, mass vaccination, regular screening of animals, and culling the positive animals are highly recommended. These are very effective in controlling and decrease the rate of the disease and risk factors. Future studies with emphasis on molecular methods are proposed to determine circulating strains in the region.

## Authors’ Contributions

MA and JG: Conceived, designed, and supervised the study. JG and ZV: Collected samples. JG, MRA, and SA: Performed the laboratory procedures. SK and AS: Analyzed the data and edited the final manuscript. All authors have read and approved the final manuscript.
